# An Inquiry into the Influence of Physical Causes upon the Moral Faculty

**Published:** 1839-02-23

**Authors:** 


### BIBLIOGRAPHICAL NOTICE.

*An Inquiry into the Influence of Physical Causes upon the Moral Faculty. Delivered before a meeting of the American Philosophical Society, held at Philadelphia, on the 27th of February,* 1786. By Benjamin Rush, M. D. Philadelphia: Haswell, Barrington, and Haswell. 1839. pp. 28.

This Address, written fifty-three years since, is again introduced to the public, under the auspices of Mr. George Combe, of Edinburgh,—now in our city, engaged in the delivery of a phrenological course,—whose object will be understood on the perusal of the subjoined well-written Introductory Notice:—

"In the numerous discussions which have arisen out of Dr. Gall's discovery of the functions of the brain, many attempts have been made to show that his views were not original. The divisions of that organ into different compartments, and the location in these of different mental faculties, exhibited by various authors, from Aristotle down to John Baptista Porta who published in the seventeenth century, have been confidently referred to, as evidences that Dr. Gall's doctrines are the mere revival of exploded theories. Dr. Gall himself has recorded the opinions and speculations of these authors, and pointed out that, while they located the faculties in different parts of the brain from fancy, he did so from observation. But the nearest approach to Dr. Gall's discovery, which has come under my notice, is one that the opponents of Phrenology have not referred to. It is contained in 'An Inquiry into the influence of Physical Causes upon the Moral Faculty,' delivered by Dr. Benjamin Rush, before a meeting of the American Philosophical Society, held at Philadelphia, on the 27th of February, 1786, published by their request, and dedicated to Dr. Benjamin Franklin. In this Inquiry 'coming discoveries' may be said to have cast their shadows before; and Dr. Rush, by observing and faithfully recording the phenomena of nature, has brought to light several important truths which have since been confirmed and elucidated by Phrenology, in a manner that evinces, on his part, extraordinary depth and perspicuity of intellect, combined with the highest moral qualities. The 'Moral Faculty' mentioned in his 'Inquiry,' appears to me to comprehend nearly the three moral sentiments of Benevolence, Veneration, and Conscientiousness, treated of by Phrenologists, each of which is manifested by means of a particular organ, and is influenced by its condition of health or disease; and if the following pages be perused with this explanation in view, the close approximation of Dr. Rush's remarks to the doctrines of Phrenology, will be easily recognised. In many details he differs from, and falls short of the views of Phrenologists; but in the general conclusion maintained by him, that physical causes influence the moral faculty, the coincidence is complete. I have not been able to find this 'Inquiry' printed separately from Dr. Rush's general works; and as it will probably prove interesting to many persons who are not in possession of these volumes, I have been induced to present it in this form to the citizens of the United States. Although all the views contained in it may not have been supported by subsequent investigations, there is so much of sagacity in the author, and of truth in his conclusions, that America may be justly proud of the genius of her son."

